# A Person-Centered Approach to the Job Demands–Control Model: A Multifunctioning Test of Addictive and Buffer Hypotheses to Explain Burnout

**DOI:** 10.3390/ijerph17238871

**Published:** 2020-11-29

**Authors:** Mafalda Gameiro, Maria José Chambel, Vânia Sofia Carvalho

**Affiliations:** CICPSI, Faculdade de Psicologia, Universidade de Lisboa, 1649-013 Lisboa, Portugal; mafaldamcgameiro@gmail.com

**Keywords:** demands–control model, engagement, burnout, latent profile analysis, person-centered approach

## Abstract

This study used a cross-sectional design and a person-centered approach in order to test the addictive and interactive strain hypotheses of Job Demands–Control Model to explain burnout. A large sample (*n* = 6357) of Portuguese workers (nurses, bank employees, retail traders, and contact center agents) was used. Through latent profile analysis (LPA), first latent profiles of demands and control were identified and then it was examined how these profiles differed in workplace well-being (engagement and burnout) through an ANCOVA. The four hypothesized profiles (i.e., “high-strain”, “low-Strain”, “passive”, and “active”) and one more profile denominated “moderate active”, emerged from LPA analysis. The hypotheses were supported in both addictive effects and interactive effects (buffer hypothesis), suggesting that the difficulty in finding consistent support for the buffer hypothesis might be related to the use of variable-centered approaches. Moreover, this reinforces that, in organizational practice, job control is a crucial characteristic to face job demands, as job control will buffer job demands’ harmful effects on workplace well-being.

## 1. Introduction

Employee’s stress and well-being are two very concerning topics either for organizational practitioners as well as researchers all around the globe. The Occupational Health Psychology field emerged in the 90s [1] and a significant amount of research in this area has shown that employee’s stress and well-being have a direct impact on many employee’s attitudes and behaviors such as satisfaction, commitment, absenteeism, turnover, performance, and extra-role behaviors [2,3,4]. Thus, it becomes of the most relevance to understand these concepts at their fullness, in order to develop new sharp theoretical knowledge and interventions that seek to improve workers and their respective companies’ job experiences and results. 

According to the well-known Job Demands–Control Model (JDC) [5,6], job demands, and job control are two key job characteristics that are strongly predictive of employees’ well-being levels. This model has the central hypothesis, that refers to how job demands and control influence directly or interactively strain (e.g., burnout) and well-being (e.g., engagement). These assumptions have already been tested several times and the support found was evident for the addictive strain hypothesis but not so much for the interactive strain hypothesis (known as buffer hypothesis) [7,8]. Mauno, Mäkikangas, and Kinnunen [9] pointed out that one possible explanation for the lack of support found for the buffering strain hypothesis was methodological due to the use of variable-centered approaches in the majority of studies. Person-centered approaches are known to be better for testing interactive frameworks as they allow to study multiple observed measures together, as a whole [10] and assume that the association between those variables can be explained by the existence of latent profiles [11].

Thus, our main objectives are to check if (1) the profiles emerge consistently with Karasek’s model assumptions, (2) if the addictive strain hypothesis is supported, and (3) if there are new findings of the buffer strain hypothesis, using latent profile analysis (LPA) and a sample with a wide range of stressful occupations. Our study adds on previous studies, a sample with a very large number of employees (i.e., 6357) from different activity sectors and occupations (health, bank, retail, and contact center). This sample variety is important not only because we expected that the results provide conclusions possible of generalization of main assumptions of JDC to a wide range of occupational sectors, but also, because these occupations are in unique work environments largely acknowledgeable as stressful [12,13,14,15,16,17,18,19,20,21]. It is in this stressful work environment that the job control may present more expression in mitigating the job demands effect, that is, that buffer hypothesis may occur [7,8].

Furthermore, understanding these relationships between job characteristics (job demands and job control) and workplace well-being (specifically, engagement and burnout) will contribute to verify the adequacy of propositions of JDC and, more specifically, to give consistence to the existence of the buffer hypothesis that is still under discussion [9]. This is an important issue to figure out in occupational stress since it provides avenues for the investment of job redesign that prevents strain, namely burnout. Indeed, this study will allow designing multidisciplinary interventions that help employers to improve their employees’ well-being, and consequently enhance their positive work outcomes and reduce the presence of the existent negative ones. Finally, this study contributes for the few existent literature that draws on a person-centered approach regarding these topics and also calls on the employment of this methodology.

### Theoretical Framework

Lazarus and Folkman [22] introduced the cognitive appraisal perspective which states that an emotional response is triggered depending on how someone evaluates the implications, meaning, or significance of some aspect of their environment. Individuals may evaluate a situation by the impact it has on their personal well-being (primary appraisal) and by the possibility of doing something to cope with the potential benefits or threats (secondary appraisal). In line with this, job characteristics can be seen as stressors as they depend on the way people perceive them. Consequently, different cognitive evaluations regarding these characteristics may influence the way job control and job demands are noticed at work. The perceptions of these characteristics strongly influence the well-being felt by employees [6].

Among many other models that were conceptualized, used as theoretical foundation, and empirically tested regarding this theme, the JDC Model [5,6] is considered one of the most well-studied and prestigious models used to research and intervene on the areas previously mentioned [23,24]. Particularly, the JDC was and still is crucial for understanding the relationships between job characteristics and their impact on employee´s well-being, burnout, health, and performance. The model showed, in several meta-analyses and reviews, an undeniable predictive validity in respect to numerous stress-related outcomes [2,25,26,27,28]. However, the majority of these studies used a variable-centered approach which could have some limitations when studying possible interaction effects [29]. Considering that, it is possible that the lack of support found for the interaction proposed by Karasek (explained ahead) was also methodological. Moreover, to better understand how demands and control interact in the same individual, it is important to consider that interaction through profiles, namely using a person-centered approach.

In person-centered approaches, the focus stands on identifying latent subpopulations of individuals relevant to the problems under study, based on multiple significantly different observed measures [10] and studying them together as a whole. This perspective sees individuals in a more holistic view [30], through some type of pattern-oriented approach [31] instead of emphasizing the separate variables [29]. Due to this reason, we chose to use LPA analysis to retest JDC model hypotheses. LPA focuses on sorting individuals into groups of individuals who are similar to each other and different to people in other groups [32] based on their patterns of observed characteristics [33]. LPA also assumes that the association between variables can be explained by the existence of latent profiles [11]. The advantages of this technique are the possibility of evaluating more rigorous criteria to determine the preferential number of profiles [30], the possibility to readily compare different models [32] and the fact that its profiles are empirical [10]—instead of chosen through cut-off scores. As so, adopting a person-centered approach, through LPA, helps us consider the combination of attributes (in this case, job demands and job control) that might usefully describe the person [34].

According to the JDC model, there are two key job characteristics that help define the psychological work environment: job demands and job control [5,6]. Although job demands started to compose some more aspects over time, such as role conflicts, physical and emotional demands [35], as well as task requirements [6], the most traditional and straightforward operationalization was made in terms of the quantitative aspects: workload and time pressure [5]. In line with Hobfoll [36], job demands can also be grasped as a perceived lack or potential loss of personal resources to cope or deal with the work environment. For example, when talking about workload demands this resource perspective proposes that those demands are stressful as an individual perceives that they have insufficient time or personal skills to complete the tasks demanded. Job control is usually operationalized as work autonomy and decision authority [37,38] as it refers to the potential control over tasks, for example, timing and method control [8] as well as conduct during the workday [5]. Rothbaum, Weisz, and Snyder [39], added the concept of “primary control” and they defend that those two aspects are the ones that allow the person to intervene directly in order to change their environment. In compliance with Hobfoll’s theory [36], job control is as a job resource that allows an employee to deal with workplace demands.

Karasek defended that the combination of different levels (high or low) of these two job characteristics (job control and job demands) resulted in four groups of perceived work environments: low-strain, high-strain, passive, and active. Low-strain group is characterized by the combination of not very demanding tasks and a very good control latitude and freedom of decision about their schedule. On the contrary, the high-strain group refers to very demanding and complex jobs with very little control. Passive jobs are undemanding jobs with little or no decision latitude (e.g., repetitive jobs). On the other hand, active jobs are highly demanding and also allow the employee to decide when and how they do their work.

Although the jobs used as samples in this paper typically are described as jobs with medium to high levels of demands, it is possible to expect the emersion of a “low-strain” group as well as a “passive job” group as there are differences between the ways people interpret and perceive their environment [22]. Thus, we established:

**Hypothesis**  **1 (H1).** *According to JDC model four different profiles of job demands and control emerge: (1) a “high-strain profile” where employees score high on demands and low on control; (2) a “low-strain profile” with low or moderate demands and high control scores; (3) an “active job profile” in which employees score both high in demands and control; (4) a “passive job profile” with low demands and control.*

The JDC model is known for its central hypotheses that concerns the relationship between the demands and control levels and the positive or negative states experienced at work. Burnout is considered the negative state and is characterized by emotional exhaustion (i.e., the draining of emotional resources), cynicism (i.e., a negative, callous, and cynical attitude towards one’s job), and lack of professional efficacy (i.e., the tendency to evaluate one’s work negatively) [40]. Burnout has negative consequences for individuals, such as, depression [41], and to organizations, such as, lower performance often leading to several forms of withdrawal [42], such as absenteeism and intention to leave the job [43,44]. On the other hand, engagement is considered the positive state of energy and connection experienced at work in relation to mental well-being and health. Due to its persistent and extensive affective-cognitive state, that is not focused on any particular thing (e.g., an object, event, person), it can also be considered as a mood, more than a simple momentary, specific emotional state. Work engagement is defined as “a positive, fulfilling and work-related state of mind that is characterized by vigor, dedication and absorption” [45]. Vigor involves high levels of energy and mental resistance, persistence when faced with difficulties and desire to invest effort in work. Dedication refers to being heavily involved at work and experience feelings of significance, enthusiasm, pride, inspiration, and challenge towards the work. Absorption is characterized by being totally concentrated and happily engrossed in one’s work and it provokes the perception that time flies when you are working [46]. Practically speaking, work engagement has become a relevant topic for organizations and their management due to its links with performance and other positive indicators such as affective commitment and extra role behavior [47].

Considering this, the central principle of the JDC model is that demanding jobs that afford little control over work are most likely to lead to decrements in well-being and to induce strain [7]. Karasek [5] started this line of thought proposing the addictive strain hypothesis that states that the most negative psychological well-being and strain levels are found in employees working in high demands and low control environments (“high-strain” jobs). This additive effect of the JDC, specifically the negative association of job demands with well-being and the positive association of job control with well-being, have received considerable support [48]. For instance, Van der Doef and Maes [28] found support in 58% of the reviewed studies and Häusser et al. [8] found support in 60% of theirs. Even though it was concluded that the evidence on the additive effects is already strong enough [8], as we are trying a different methodological approach, we propose that:

**Hypothesis**  **2 (H2).** *The “high-strain profile” is associated with worse levels of well-being (low engagement and high burnout) than the other profiles.*

In contrast, the buffer hypothesis refers exclusively to an interactive effect of both demands and control in well-being. A buffering effect is a process where a psychological resource reduces the impact of stressors on psychological well-being. In this case, in line with Hobfoll’s theory [36], job control is seen as a resource that contributes to adjustment and is predicted to attenuate the negative impact of demands on well-being [5,6]. In other words, control acts as a moderator on the negative relationship between job demands and well-being since autonomous employees actually intervene and actively change their job conditions [49]. By having control over their work, employees conduct their work tasks, restructure their pacing and timing and choose from different methods to accomplish their working goals [50], reducing the perception of insufficient or potential loss of personal resources [36]. According to Fila [7], not only this interactive proposition is intuitively attractive, but it is also consistent with a broad assortment of other general theoretical and primary research on the importance of control in reducing stressors’ effects. However, Karasek himself, who proposed and examined this interaction effect in 1979, stated later, in 1989, that the existence of this effect was not the primary issue of his model. Moreover, Carayon [51] and Jones and Fletcher [4] found it difficult to demonstrate empirical support for this interaction in burnout prediction. Furthermore, overall empirical support for the interactive/buffering effects of the JDC model is a lot less consistent than the support for the additive effects of the strain hypothesis. For instance, Van der Doef and Maes [28] found that interaction effects were supported only in 48% of studies, often just partially. In Taris’ words [52], they found that this interaction depended on participants’ scores on third variables (e.g., personality traits) or on the type of analysis conducted (e.g., ANOVA versus regression analysis). The review by Häusser et al. [8] found that the corresponding proportion was just 39% and pointed the samples and the measure-based differences as the possible reasons for this lack of support. Mauno et al. [9] also argued that one reason may be methodological once the majority of approaches taken were variable-centered by computing an interaction term (demands × control). Considering this raised limitation, and as said before, we chose to use LPA, in order to retest this possible interaction using a person-centered approach. Specifically, the emergence of a profile tells us how the job characteristics (demands and control) in individuals are being combined, that is, how they interact. Thus, verifying the effects on the well-being of a profile where there is an interaction of high demands and low control (“high-strain profile”) compared to a profile where there is an interaction of high demands but also of high control (“active work profile”), will allow us to conclude about the mitigation effect that control produces on the negative consequences of job demands, namely on burnout.

Positive findings concerning this interaction are relevant due to the fact that, if found, they will support the need to raise employees’ job control, without having to reduce demands, in order to improve well-being (i.e., reduce burnout and promote engagement). As De Jonge, Dollard, Dormann, Le Blanc, and Houtman [53] pointed out, this would be a significant finding once there is a persistent difficulty in reducing demands if organizations want to progress on the competitive global market. On the contrary, if only the additive strain hypothesis keeps being supported, this strategy would not be effective once job demands would maintain their damaging effect on employees’ well-being. 

For the previously stated reasons, we aim to re-analyze, using a latent profile analysis, whether or not control buffers the negative consequences of demands (burnout) and maintains the presence of positive work well-being (engagement). Therefore, we propose that:

**Hypothesis** **3 (H3).** *The “active profile” is associated with better levels of well-being (high engagement and low burnout) than the “high-strain profile”.*

## 2. Method

### 2.1. Participants and Procedure

The sample used in this study consisted of 6357 Portuguese employees from four different occupations: nurses (*n* = 858), bank employees (*n* = 1769), retail traders (*n* = 922), and contact center agents (*n* = 2808). Approximately, 64% of the sample were women (*n* = 4067). Data were collected via electronic questionnaires sent by the management departments of each company which called on its voluntary participation. All participating organizations received an e-mail containing the link to the survey, and the companies forwarded the e-mail to all their employees. Participants filled the questionnaire online, through SurveyMonkey’s platform, during working hours and without any compensation associated. Anonymity of the answers was assured. The participants were informed of the opportunity to receive feedback on the overall results and that organizations would have access to the final report and not to the data itself. Given that the collaboration was voluntary and anonymous, there was no need for the participants to sign an informed consent form. The participant response rates ranged from 56% to 83%, depending on the organization from which the data originated. The faculty’s local ethics committee (Faculdade de Psicologia da Universidade de Lisboa) granted ethical approval for this study.

### 2.2. Measures

*Job characteristics* were measured using a Portuguese version [54] of the Job Content Questionnaire [35]. There were seven items for job demands (e.g., “I have too much to do”; α = 0.84) and four items for job control (e.g., “I have the opportunity to decide how to organize my work; α = 0.85). Both dimensions were scored on a 5-point rating scale from 1 (totally disagree) to 5 (totally agree).

*Well-being* was measured with the assessment of work engagement and burnout. Work engagement was measured with a Portuguese version of the Utrecht Work Engagement Scale (UWES-9) [55] that included nine items (e.g., “When I wake up in the morning, I feel good about going to work”; α = 0.94). We performed a confirmatory factor analysis in order to test if the measurement model of engagement fit better with the three separate dimensions (i.e., vigor, dedication, and absorption) or by using engagement measured with a single dimension that was also used in previous studies [56]. The engagement model fit composed by the three dimensions presented a good fit (χ2 (24) = 1252.49, p < 0.01, CFI (comparative fit index) = 0.97; TLI (Tucker–Lewis index) = 0.96; RMSEA (root mean square error of approximation) = 0.09) as the model fit of engagement composed by a single dimension (χ2 (24) = 1252.49, p < 0.01, CFI = 0.97; TLI = 0.96; RMSEA = 0.09). By comparing the two models, the model with a single dimension was significantly better (Δχ2(3) = 122.08, *p* < 0.001). Thus, a single-dimension version of engagement was used. 

Burnout was measured through the exhaustion and cynicism dimensions. In spite of professional efficacy initial inclusion as the third dimension of burnout [40], this dimension has been considered a relatively independent dimension [57] that is developed in a parallel way [58]. Thus, we considered the core dimensions of burnout (i.e., exhaustion and cynicism). Exhaustion was assessed by five items (e.g., “I feel emotionally drained by my work”; α = 0.91) and cynicism was assessed by five items (e.g., “I question the significance of my work”; α = 0.82) [59]. Respondents answered the items of this scales on a 7-point scale ranging from 1 (never) to 7 (every day). These scales have previously been used in Portuguese studies [60,61].

*Control Variables*. Gender was added as control variable since previous studies underlined that it is a variable susceptible of differences in relation to job demands and job control perceptions [62]. In the same vein, we added occupation (nurses, bank employee, retail traders, and contact center agents) and if the participant has a supervisor function as control variables. 

### 2.3. Data Analysis

The statistical analysis consisted of three stages. In the first stage, a confirmatory factor analysis [63] with structural equation modeling methods was implemented with Mplus 7.2 (IBM Corp, Armonk, NY, USA) [64]. The maximum likelihood estimation provides the well-known global fit statistics for structural equation modelling methods: comparative fit index (CFI; satisfactory values of 0.90 and above), Tucker–Lewis index (TLI; satisfactory values of 0.90 and above), and root mean squared error of approximation (RMSEA; satisfactory value below 0.08) [65]. In the second stage, a latent profile analysis (LPA), to identify demands and control latent profiles [64] and test for H1. The optimal number of profiles was identified by performing two to six group solutions, starting by the model with two profiles and successively adding one until the six-profile model. Each model was evaluated according to the following parameters: the Bayesian information criteria (BIC), entropy values, and the Lo–Mendell–Rubin adjusted likelihood ratio test (LMR). The best possible profiles model should have the lower BIC, the highest entropy, a significant LMR *p*-value, and enough people in each profile.

The third stage consisted of the analysis of the other proposed hypothesis (H2 and H3). We used BM Statistical Package for the Social Sciences (IBM Corp, Armonk, NY, USA) to conduct a one-way analysis of covariance (ANCOVA) followed by post hoc comparisons that used the profile group as the independent variable. Gender, occupation, and supervision function were included as control variables.

## 3. Results

### 3.1. Correlations between Variables

Analyzing the correlations among the studied variables (Table 1), job characteristics are significantly related with employees’ well-being in a way that demands have a negative relationship with engagement and a positive relationship with exhaustion and cynicism. On the other hand, control has a positive relationship with engagement and a negative relationship with exhaustion and cynicism. 

### 3.2. Confirmatory Factor Analysis

The goodness-of-fit index of the theoretical model (i.e., demands, control, engagement, and burnout, where the second-order latent variable was explained by exhaustion and cynicism) presented an acceptable fit to the data: χ^2^ (395) = 12,947.91, *p* < 0.01, CFI = 0.91; TLI = 0.90; RMSEA = 0.07). We compared this model with one single factor model where all items loaded on a single latent variable. Our analysis showed a significantly poorer fit on the single factor model χ^2^ (405) = 60,188.10, *p* < 0.01, CFI = 0.56; TLI = 0.52; RMSEA = 0.15). In that sense, our theoretical model presented the best fit to the data (Δχ^2^(10) = 47,240.19, *p* < 0.001).

### 3.3. The Demands and Control Profiles

LPA was used to identify latent profiles of job demands and job control. We tested six profile solutions and compared BIC, entropy, LMR values, and number of people in each profile. Although the six-profile solution had a lower BIC value and higher entropy 0.76, it presented one profile with only 41 employees which are not representative considering the total number of people in the sample (*n* = 6357). Therefore, we considered that the five-profile solution had the best fit (Table 2) presenting lower BIC value comparing with the other (two, three, and four) profile solutions, a good entropy value, a significant LMR *p*-value, and a considerable number of people in each profile (profile 1 *n* = 115; profile 2 *n* = 334, profile 3 *n* = 872; profile 4 *n* = 4232; profile 5 *n* = 803).

**Figure 1:**
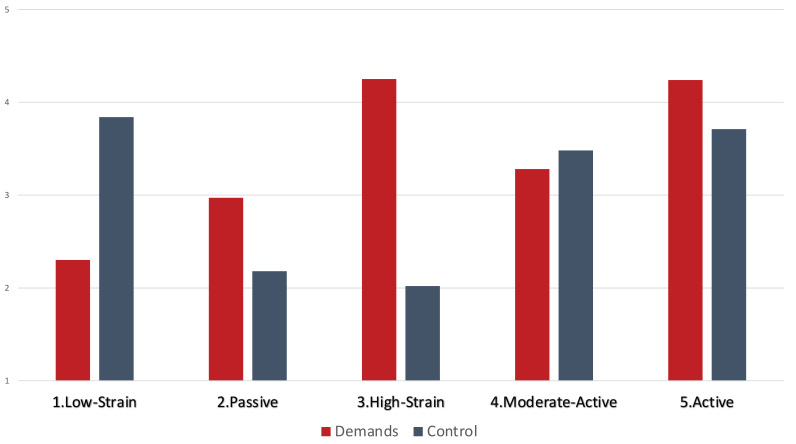
Demands and Control Profiles.

Table 3 and Figure 1 show the results for the five-profile solution. Participants in the first profile (*n* = 115), which we called “low-strain profile” had moderate levels of demands (2.30) and high levels of control (3.84). The second profile (*n* = 334), had low-moderate levels of demands (2.97) and control (2.18) and we called it “passive profile”. The third profile (*n* = 872) presented high levels of demands (4.24) and low levels of control (2.02), and thus, we called it the “high-strain profile”. The fourth profile, the “moderate active profile” (*n* = 4232), comprising the major part of the sample, was characterized by medium levels of demands (3.28) and medium-high levels of control (3.48). Finally, the last profile (*n* = 803) was labeled the “active profile” for high levels of demands (4.24) and high control (3.71). Thus, the four profiles designated in hypothesis 1 emerged and an additional profile emerged—“the moderate active profile”. Thus, we consider the H1 was partially supported. 

### 3.4. The Addictive Hypothesis

Table 4 shows the mean values of engagement, exhaustion, and cynicism in each profile. The “high-strain profile” reported the lowest engagement (3.95) and the highest exhaustion (3.82) and cynicism (3.53). The comparison of these variables between all the profiles was significant (*p* < 0.001; Table 4). Thus, these results are in line with JDC addictive hypothesis and support H2.

### 3.5. The Buffer Hypothesis

Regarding the buffering hypothesis, by observing Table 4, we can observe that the “active profile” presents the better well-being levels (high engagement (5.10) and low burnout (4.3—exhaustion; 2.74—cynicism) compared with the “high-strain profile”. Accordingly, these results are in line with JDC buffer hypothesis and support H3. 

## 4. Discussion

This study was set out to analyze the presence of demands and control profiles in a large Portuguese database composed by four distinct occupations (i.e., nursing, bank employees, retail traders, and contact center agents) in order to retest JDC addictive and buffer strain hypotheses. Latent profile analysis to test the emergence of profiles was used, and then, the relationship of each profile with workplace well-being (i.e., burnout and engagement) was analyzed. 

The identified profiles were congruent with the previous literature concerning the expected balance between demands and control. In line with JDC model and what was hypothesized, we could find four profiles—a “passive profile” (low demands and low control), a “low-strain profile” (low demands and high control), a “high strain profile” (high demands and low control), and an “active profile” (high demands and high control). Was also observed a “moderate active profile” (moderate demands and moderate control). The study of Mauno et al. [9] also used a person-centered methodology to detect profiles in a sample of Finnish university employees and detect the emergence of four profiles that match with the ones described, with exception to the passive profile and to the moderate active profile that Mauno et al. [9] did not find. However, it is important to note that the Mauno et al. [9] study analyzed the changing nature of the profiles over time and verify that two of the profiles change over time in relation to job control. They found that the low-strain profile and high-strain profile were stable over time and another two profiles founded vary in terms of job control (one that job control increases and other where job control decreases). We may infer that the two unstable profiles correspond to our active and moderate active profiles, although with caution since our study is cross-sectional. As Mauno et al. [9] also stated based on previous research [5,66] and their own findings, this may signify that the high and low strain profiles are universals. Our results bring strength to this idea. 

Regarding the prevalence of participants in each profile, is important to note that we observed that both profiles characterized by low demands, i.e., low-strain and passive, included a lower number of workers. This might be due to the nature of the occupations in the sample (nursing, bank employees, retail traders, and contact center agents) that have been known for generally being high-strain occupations [67], characterized by high workloads and a lot of time pressure. In line with this idea, we also observed that the majority of workers were included in the profiles that have high or moderate job demands. Contrary, the study of Mauno et al. [9] had found the majority of prevalence in the low-strain profile, characterized by low demands. This fact justifies the necessity to pursue with studies that cover a wide number of professional sectors to detect the profiles of JDC model emergence.

Concerning the JDC addictive hypothesis, it was supported since, as expected, the high-strain profile—characterized by high demands and low control—reported the lowest engagement and the highest burnout. It is important to note that a large amount of research [8,26,28,48] already stated the well-known background for this central tenet of JDC. However, regarding the method of analysis, studies frequently used the multiple regression analysis or structural equation models [26]. By using a method that searches to find a pattern-oriented approach—profiles—our results continue to support the JDC addictive strain hypothesis. 

More interesting, by using the LPA as a person-centered approach, we also found support for the buffer hypothesis—job control buffers the relationship between job demands and strain/well-being since by comparing the “active profile” and the “high-strain profile” (both with high job demands) that emerge in our multifunctional sample, the one with better well-being levels was the active, the one with high control. This result is relevant because, as previous studies underlined [8,28,52], the interaction between demands and control has received less support in the literature. Hence, the results of this study highlighted the crucial role that job control may have on the relationship between job demands and workers’ well-being. This is extremely relevant as it indicates that, independently from job content that derives from specific occupations such as nursing, bank employees, retail traders, and contact center agents, when designing interventions and thinking about employees’ well-being, there is no crucial need to decrease job demands if control over work is provided.

Overall, we may conclude that the JDC model was supported in this setting with respect to profiles emergence and their relationship with workplace well-being, namely burnout and work engagement.

### 4.1. Study Limitations and Future Research

This study presents a few noteworthy limitations. First of all, all data was collected through self-reported measures, thus, it is possible that common method bias may have affected the results. However, as we used scales and models that have already been used and tested for several times, we believe that the risk is smaller. Furthermore, when working with self-reported measures, and even if disclosure and confidentiality are secured, participants may respond according to social desirability. Second, being a cross-sectional study, it does not allow to infer causal relationships but only their direction (positive or negative) and significance. Thus, we propose the replication of the study but with several measures of the variables, over time (longitudinal design). Third, on the revised JDC model (JDCS model) [6] social support is included as another key job characteristic. We opted to not include this variable due to practical reasons as it would make the models even more complex to analyze. Even though, we believe that including this variable in the future would bring more interesting aspects to our study. Fourth, we did not have outcome variables to test the learning hypothesis (e.g., a scale measuring skill learning). Thus, we propose that those future studies include this outcome and also test this hypothesis of the JDC model. In general, our study gives consistency to the hypotheses initially advocated in the JDC. Specifically, with regard to the buffer hypothesis, we warn that it should not only continue to be investigated in samples with different occupations, but also with regard to its specificity. For example, Mauno et al. [9] warn of how autonomy can be perceived in different ways (e.g., working hours, decision making), and these different forms of autonomy may also have a unique role in the buffer hypothesis, which is why we also recommend this investigation in future studies. Further, the study of Topcic, Baum, and Kabst [68] suggested that participating in decision-making might increase stress among employees. As we underlined, the ways people interpret and perceived their environment [22] are determinant. Thus, we recommend that future studies explore the JDC assumptions taken into consideration variables that within the organizational context may influence the way people perceive job characteristics, such as, the organizational culture, leadership styles, or team climate. Moreover, individual factors such as socioeconomic status and personality variables (e.g., self-efficacy, locus of control), have been related to a predisposition to burnout and may interfere in the buffer hypothesis [7,69]. Thus, these variables should also be taken into account in future studies. Job demands should also be explored more deeply since the literature provide evidence that they may be divided into hindrance (e.g., administrative frustration, resource inadequacy, role conflict, excessive responsibilities, and time urgency) or challenge demands [70]. Thus, the buffer hypothesis should be explored regarding different demands and different control types. In addition, we believe that more investigations using longitudinal person-centered approaches will be useful to keep on funding support for the hypothesis presented in this study.

#### Practical implications

Despite the aforementioned limitations, our study provides evidence that JDC in its simplistic form continues to forefront occupational well-being. Thus, with a methodologically different approach (LPA) and with a sample with a wide range of occupations, typically characterized by stressful occupations [12,13,14,15,16,17,18,19,20,21], our study highlights that redesign jobs through the two main job characteristics have impact on employees’ burnout and engagement. The most salient aspect of the current study is that organizations should invest in workers’ control. Depending on the occupational sector, different forms to provide control may develop. Generally, we emphasized that rearranging the work process so that individual workers or subgroups of workers can control the pace of work and execution of tasks, to create autonomous work teams by consigning each team the responsibility to decide how to perform the work, to organize group discussions to think about work processes, taking into account the proposed ideas and other practicable options, are good examples of measures that can be taken for different professional sectors. Furthermore, supervisors may play a critical role in helping workers to set priorities for day-to-day work, set goals, decide how to perform work, and select approaches to doing work and deciding how to improve their tasks.

## 5. Conclusions

This study provides support for addictive and interactive effects (buffer hypothesis) in a Portuguese database composed by four distinct occupations (i.e., nursing, bank employees, retail traders, and contact center agents). Latent profile analysis shown the emergence of five different profiles with different configurations of demands and control. The relationship of each profile with burnout and engagement highlighted the importance that demands’ and control have to employees’ well-being and, in particular, is reinforced the role that control have not only in preventing ill-being but also in promoting well-being.

## Figures and Tables

**Table 1:** Means (M), standard deviations (SD), and correlations (r) of the study variables.

Study Variables					*r*			
	*M*	*SD*	1.	2.	3.	4.	5.	6.
1. Function	2.59	0.89						
2. Supervision function	0.19	0.40	−0.20 **					
3. Demands	3.51	0.74	−0.08 **	0.15 **				
4. Control	3.23	0.86	−0.19 **	−0.22 **	−0.14 **			
5. Engagement	5.05	1.39	−0.06 **	−0.16 **	−0.23 **	0.45 **		
6. Exhaustion	3.57	1.65	−0.03 **	−0.08 **	0.44 **	−0.30 **	−0.52 **	
7. Cynicism	2.49	1.41	0.09 **	−0.12 **	0.27 **	−0.36 **	−0.58 **	0.64 **

*Note*: ** *p* < 0.01. Function and supervision function are codified as dummy variables.

**Table 2:** Fit indices for the six estimated solutions of job demands and job control profiles.

Profile Number	BIC	Entropy	LMR *p* Value
2	29,878.92	0.72	**
3	29,863.54	0.66	*n.s.*
4	29,838.50	0.59	**
5	29,800.08	0.65	**
6	29,739.75	0.76	**

*Note.* ** *p* < 0.001; *n.s.* = nonsignificant; BIC: Bayesian information criterion; LMR: Lo–Mendell–Rubin test.

**Table 3:** Mean values for job demands and job control in each identified profile.

Profiles	Job Demands *M*	Job Control *M*
1. Low-Strain	2.30	3.84
2. Passive	2.97	2.18
3. High-Strain	4.25	2.02
4. Moderate-Active	3.28	3.48
5. Active	4.24	3.71

**Table 4:** Mean values of demands, control, engagement, exhaustion, and cynicism for each of the five profiles.

Profiles	Engagement *M*	Exhaustion *M*	Cynicism *M*
1. Low-Strain	5.68	2.04	1.81
2. Passive	4.56	3.44	2.78
3. High-Strain	3.95	4.82	3.53
4. Moderate Active	5.30	3.22	2.23
5. Active	5.10	4.34	2.74
Bonferroni pairwise comparisons between profiles	1 > 4 > 5 > 2 > 3	3 > 5 > 2 > 4> 1	3 > 2 > 5 > 4 > 1
